# Applying a data-driven population segmentation approach in German claims data

**DOI:** 10.1186/s12913-023-09620-3

**Published:** 2023-06-08

**Authors:** Carolina Pioch, Cornelia Henschke, Hendrikje Lantzsch, Reinhard Busse, Verena Vogt

**Affiliations:** 1 Department of Health Care Management, Technical University of Berlin, Straße des 17. Juni 135, Berlin, 10623 Germany; 2 Berlin Centre of Health Economics Research (BerlinHECOR), Technical University of Berlin, Straße des 17. Juni 135, Berlin, 10623 Germany; 3 Institute of General Practice and Family Medicine, Jena University Hospital, Friedrich Schiller University, Bachstraße 18, Jena, 07743 Germany

**Keywords:** Population segmentation, Healthcare utilisation, Population health, Cluster analysis, Claims data

## Abstract

**Background:**

Segmenting the population into homogenous groups according to their healthcare needs may help to understand the population’s demand for healthcare services and thus support health systems to properly allocate healthcare resources and plan interventions. It may also help to reduce the fragmented provision of healthcare services. The aim of this study was to apply a data-driven utilisation-based cluster analysis to segment a defined population in the south of Germany.

**Methods:**

Based on claims data of one big German health insurance a two-stage clustering approach was applied to group the population into segments. A hierarchical method (Ward's linkage) was performed to determine the optimal number of clusters, followed by a k-means cluster analysis using age and healthcare utilisation data in 2019. The resulting segments were described in terms of their morbidity, costs and demographic characteristics.

**Results:**

The 126,046 patients were divided into six distinct population segments. Healthcare utilisation, morbidity and demographic characteristics differed significantly across the segments. The segment “High overall care use” comprised the smallest share of patients (2.03%) but accounted for 24.04% of total cost. The overall utilisation of services was higher than the population average. In contrast, the segment “Low overall care use” included 42.89% of the study population, accounting for 9.94% of total cost. Utilisation of services by patients in this segment was lower than population average.

**Conclusion:**

Population segmentation offers the opportunity to identify patient groups with similar healthcare utilisation patterns, patient demographics and morbidity. Thereby, healthcare services could be tailored for groups of patients with similar healthcare needs.

## Background

Empirical approaches to population segmentation are becoming increasingly important internationally, especially in developing effective, patient-centred care concepts for an entire population [1]. In this context, personalised therapy, patient education, and empowerment as well as shared decision-making have been promoted in many Western countries [2, 3]. However, due to limited resources both in terms of time and costs, healthcare services should be tailored to patient groups with similar patterns of healthcare needs rather than to individuals in the population [1, 4]. Segmenting the population into relatively homogenous groups according to their healthcare needs may help to get a better understanding of the population’s demand for healthcare services and thus enhance the allocation of healthcare resources and the planning of interventions [4–6].

Currently, two approaches are used for population segmentation. In expert-driven approaches, a population is segmented by an expert panel based on pre-determined criteria derived from literature review and consensus [7]. For example, the “Bridges to Health model” developed by Lynn et al. stratifies the entire population into eight segments based on health prospects and priorities. At any point in their life, the people fit into one of these segments which comprise the full range from healthy patients to dying patients who are rapidly deteriorating [4]. However, using medical criteria to group populations does not adequately represent the actual healthcare utilisation. An alternative are data-driven approaches, in which various statistical methods are applied post-hoc to large population datasets to profile patient segments [7].

There are a few previous studies that performed a data-driven approach on a patient population [5, 6, 8]. Vuik et al. used a random sample of 300,000 patients in England and grouped them into segments based on the following utilisation variables: non-elective inpatient admissions, elective inpatient admissions, outpatient visits, general practitioner (GP) practice visits, GP home visits, and prescriptions [5]. The same approach was adapted by Nnoaham et al., who replaced GP home visits by emergency department visits as a segmentation variable to divide a population of 80,000 people in one geographically defined region in England [6]. In addition to the utilisation variables, Low et al. included age as a demographic variable to determine patient segments from 150,000 patients in the Singapore Health Services Regional Health System and examine their predictive ability for healthcare utilisation and mortality longitudinally [8]. In all studies, different segments with distinct healthcare utilisation patterns were identified and used to generate targeted healthcare interventions for each segment [5, 6, 8].

Tailoring interventions for segments with similar healthcare needs may improve coordination of care in the highly fragmented health system of Germany which is due to different legislation, planning and regulation for ambulatory primary and specialised care, inpatient care and long-term care [5, 6, 9]. In Germany, there is no traditional gatekeeping in outpatient care. Patients can freely select their physicians, GPs and specialists alike. Ambulatory primary care provided by GPs and ambulatory secondary care provided by specialists both take place in physicians’ practices and medical care centres outside hospitals. Some ambulatory care services are also delivered in hospitals to encourage cross-sectoral and multidisciplinary treatment for diseases that require specialised equipment. Ambulatory hospital care services comprise highly specialised care and services as well as minor surgeries [9].

So far, no population segmentation has been performed based on German claims data. Previous studies focused on grouping patients with a certain disease, e.g., Schäfer et al. examined reasons of not participating in disease-specific educational programs among patients with diabetes. Their cluster analysis was based solely on data extracted from electronic medical records of the patients’ GPs while data on the utilisation of services in various healthcare settings were missing [10]. Our study is the first to apply a data-driven utilisation-based segmentation approach on an entire population dataset in a defined region in Germany. By using German claims data, the utilisation of healthcare services in a region can be comprehensively identified as all residents in Germany are legally required to have either statutory or private health insurance, with statutory health insurance funds covering about 90% of the population. There are no further costs for the insureds, apart from small co-payments of e.g., 10 euros per inpatient day of a hospital stay [9, 11]. The aim of the explorative study was (1) to identify segments of patients with different healthcare utilisation patterns using cluster analysis and (2) to discuss the results in the light of the results of previous studies mentioned above.

## Methods

### Data

The data analysis was carried out as part of a project which aimed at developing an innovative, data-supported primary care model for a rural district (“Landkreis”) in the south of Germany. Pseudonymised claims data was provided by one big German statutory health insurance fund (AOK Baden-Wuerttemberg) that covered more than 50% of the population in this district. In 2019, the rural district had a total population of about 287,000 (50.43% female), with 279 persons per square kilometre [12]. While the gender distribution was similar to the total population in Germany (50.66% female), the rural district was more densely populated compared to the German average of 233 persons per square kilometre [13]. The cross-sectional study included data on all adults (18 years of age and above) who had AOK insurance for at least one day in 2019 and resided in the rural district. For each patient, the following healthcare utilisation variables were selected: GP visits, specialist ambulatory visits, emergency visits, non-elective inpatient admissions, elective inpatient admissions, and ambulatory hospital visits. Specialist ambulatory visits were divided into general specialist care and specialised specialist care, as providers are considered in different planning schemes for the regional distribution of physicians in Germany depending on their specialisation. In total, there are four different provider groups: GPs, general specialists (e.g., ophthalmologists or gynaecologists), specialised specialists (e.g., anaesthetists or cardiologists) and separate specialists (e.g., pathologists or human geneticists). The fourth provider group, however, was not considered in the analysis as the group of separate specialists is organised on a much larger planning scale than the other provider groups (and immediate accessibility is rather secondary) [14]. Since doctor-patient contacts are covered by a per capita payment which in Germany is usually billed at the first visit in a quarter, ambulatory practice visits were considered in a quarterly period. In addition, healthcare utilisation included both visits in- and outside the defined region. Data on patient characteristics including age, gender, disease patterns, long-term care grade, residential care status and costs were also extracted (Table 1).

**Table 1:** Variables used and their definition

Variables	Definition
**Utilisation**	
GP visits	Visits at the GP’s practice and home visits by the GP. Multiple doctor-patient contacts in one quarter are counted as one visit
General specialist visits (“Allgemeine Fachärzt*innen “)	Visits at the specialist’s practice and home visits by the specialist The following disciplines are included: ophthalmologists, surgeons, gynaecologists, ear, nose and throat specialists, dermatologists, neurologists, psychologists, orthopaedists, urologists, paediatricians [14]. Multiple doctor-patient contacts in one quarter are counted as one visit
Specialised specialist visits (“Spezialisierte Fachärzt*innen”)	Visits at the specialist’s practice and home visits by the specialist The following disciplines are included: specialised internists (e.g., cardiologists, haematologists, oncologists), anaesthetists, radiologists, paediatric psychiatrists [14]. Multiple doctor-patient contacts in one quarter are counted as one visit
Emergency visits	Ambulatory emergency visits in the emergency department of hospitals and ambulatory out-of-hours visits in outpatient care according to the definition of the uniform value scale (“Einheitlicher Bewertungsmaßstab”, EBM)
Non-elective inpatient admissions	All inpatient emergency visits. All inpatient cases for which the reason “emergency” was coded
Elective inpatient admissions	All inpatient admissions for which the reason “full inpatient hospitalisation” was coded. Part time hospitalisations and admissions due to transfers from other hospital providers were excluded to avoid multiple counts (and thus an overestimation of treatment cases)
Ambulatory hospital visits	Visits where patients do not stay overnight in hospital before or after treatment (e.g., minor surgeries, highly specialised care and services) and which are billed as outpatient at the same time. Ambulatory emergency hospital visits were excluded
**Patient characteristics**	
Age in 2019	Age on the last day of being insured with the health insurance fund in 2019
Gender	Gender is classified as female or male
Residential care	Long-term care given to persons in need for care who stay in a residential setting
Long-term care grade	Long-term care grades indicate the individual need for nursing care considering physical, mental, and psychological conditions based on the extent of the limitations to independence and capabilities. Classified into one of five different care grades: minimal impairment (= 1) to the most serious impairment (= 5) [15]
Disease pattern	Based on the Charlson comorbidity index which is initially used to predict mortality in 17 clinical conditions based on International Classification of Diseases 10^th^ revision (ICD-10). The severity of comorbidity is categorised according to three grades: mild (= 1–2), moderate (= 3–4), severe (≥ 5) [16]. Includes all confirmed diagnoses in the ambulatory setting and all main and secondary diagnoses in the inpatient setting
**Costs in 2019**	Includes the net costs for the health insurance fund which occurred in the following healthcare settings: GP care, general specialist care, specialised specialist care, emergency visits, non-elective inpatient admissions, elective inpatient admissions, ambulatory hospital care

*Abbreviations: GP* general practitioner

The legal requirements for the scientific use of case-related or personal data from the statutory health insurance in Germany were applied in accordance with the Social Code Book (“Sozialgesetzbuch”, SGB). The basis for the use of the pseudonymised claims data in this study is paragraph § 75, Sect. 1, number 1, SGB X (research projects based on social data) and was approved by the responsible supervisory authority. Contractual arrangements were made for the data collection and use after approval. The contract outlined each party’s duties and rights regarding the extent (including data scope) and length (including naming of deletion periods) of use as prescribed in paragraph § 75 SGB X. In compliance with data protection laws, the AOK Baden-Wuerttemberg has provided pseudonymised data by replacing personal identifying information (in this case health insurance number) with a pseudonym.

### Segmentation variables

The variables used for segmentation included age and the seven healthcare utilisation variables described above. Age gives implications on the demand for and utilisation of health and social services [17, 18], whereas the seven healthcare utilisation variables represent the use of different healthcare providers and settings [5]. Both age and five of the utilisation variables have been used by previous studies for a data driven segmentation approach, whose segmentation method was also applied in this study [5, 6, 8]. Furthermore, the number of specialist ambulatory visits was separated into general specialist care and specialised specialist care. The number of ambulatory hospital visits was included to give an additional understanding on the population’s demand for healthcare services. Due to different scales across the variables, all segmentation variables were z-standardised by subtracting the mean of each variable and dividing it by its standard deviation.

### Segmentation method

For the segmentation of the population a combined approach was selected. First, the hierarchical cluster analysis (Ward’s method) was applied as stopping rules are readily available to determine the optimal number of clusters (k) [19]. Its aim is to minimise the cluster sum of squares and thereby maximise homogeneity within the clusters [20]. However, hierarchical cluster methods are not suitable for large datasets [21]. Therefore, it was followed by a non-hierarchical k-means clustering algorithm with a Euclidean distance which can handle large datasets in an efficient way [22].

In accordance with previous studies, Ward’s method was run on ten random subsets of 3,000 patients to define the optimal k [5, 6, 8]. For each of the subsets, two- to 15-cluster-solutions were compared by calculating the Calinski and Harabasz pseudo-F index [19] and the Duda-Hart Je (2)/Je (1) index F [23]. The Calinski and Harabasz pseudo-F index compare the between-cluster variation to the within-cluster variation, thereby assessing the cluster tightness. The Duda-Hart Je (2)/Je (1) index F uses the ratio of the two within sum of squares to decide whether the current cluster can be further split. High values of the pseudo-F index and the Duda-Hart index, with its corresponding low pseudo T-squared value and high pseudo T-squared values on either side, indicate distinct clustering and hence potential cluster solutions [19, 23, 24]. Across the ten subsets, this method resulted in four- to seven-cluster solutions. For these four k values, the k-means clustering algorithm was applied to the full dataset. Each cluster solution was evaluated by its clinical relevance and interpretability [5, 6], resulting in k = 6 to be the optimal cluster solution. To confirm that the cluster solution did not appear by random chance, a split-sample analysis was conducted [5]. The full dataset was split in two equal-sized subsets which were analysed with the k-means clustering algorithm for six clusters. The segments showed the same results as the ten random subsets. All cluster analyses were performed using R Statistical Software, including the packages cluster, clusterSim and NbClust to conduct the cluster analyses and the package comorbidity to compute the Charlson index [25].

### Statistical analysis

The segments were then analysed to identify significant differences across the segments. For the healthcare utilisation variables, a Kruskal–Wallis test was used, because they did not meet the normality assumptions. For age, a one-way ANOVA test was computed. For the variables gender, residential care status, long-term care grade and disease patterns a chi-square test was calculated.

A pairwise segment comparison was then performed on the variables which differed significantly using Mann–Whitney U tests, Student t-tests, and z-tests. Furthermore, a Bonferroni correction was conducted at the significance level of 0.05 for the pairwise tests to counteract the problems of multiple testing occurring when comparing the segments.

## Results

### Patient characteristics

As shown in Table 2, the study included 126,046 (51.85% female) patients with an average age of 50.80 years and a standard deviation (SD) of 19.72. About 4% of the study population changed their health insurance fund throughout the year. The k-means cluster analysis revealed six distinct clusters. For each cluster, a label was chosen that best reflects the insured’s healthcare utilisation in the specific segment. Thus, the following names for the segments were set: “Low overall care use”, “High primary care use”, “High emergency care use”, “High specialist care use”, “High hospital care use” and “High overall care use.” Age and all seven healthcare utilisation variables differed significantly across the six segments with *p* < 0.001, reflecting the purpose of cluster analysis to maximise the distance between the clustering variables.

**Table 2:** Patient characteristics and segmentation outcome

	**All**	**Segment 1: low overall care use**	**Segment 2: high primary care use**	**Segment 3: high emergency care use**	**Segment 4: high specialist care use**	**Segment 5: high hospital care use**	**Segment 6: high overall care use**	***p*-value**
**Number of patients (%)**	126,046 (100)	54,056 (42.89)	39,307 (31.18)	13,030 (10.34)	12,578 (9.98)	4,518 (3.58)	2,557 (2.03)	
Age in 2019 (years, mean (SD))	50.80 (19.72)	37.76 (13.02)^a^	66.58 (14.22)^a^	41.24 (17.02)^a^	62.13 (15.47)^a^	55.16 (18.31)^a^	69.01 (18.59)^a^	< 0.001
Gender (female %)	65,359 (51.85)	23,847 (44.12)^a^	22,601 (57.50)^b^	7,008 (53.78)^a^	7,973 (63.39)^b^	2,652 (58.70)^a^	1,278 (49.98)^a^	< 0.001
Residential care (%)	2,395 (1.90)	110 (0.20)^a^	1,105 (2.81)^a^	235 (1.80)^a^	78 (0.62)^a^	485 (10.73)^a^	382 (14.94)^a^	< 0.001
Long-term care grade								
None (%)	116,506 (92.43)	53,625 (99.20)^a^	34,381 (87.47)^a^	12,400 (95.17)^a^	1,1470 (91.19)^a^	3,464 (76.67)^a^	1,166 (45.60)^a^	< 0.001
1 (%)	970 (0.77)	35 (0.06)^a^	483 (1.23)^a^	51 (0.39)^b^	185 (1.47)^b^	114 (2.52)^a^	102 (3.99)^a^	< 0.001
2 (%)	3,634 (2.88)	171 (0.32)^a^	1,900 (4.83)^a^	198 (1.52)^b^	565 (4.49)^b^	409 (9.05)^a^	391 (15.29)^a^	< 0.001
3 (%)	2,715 (2.15)	114 (0.21)^a^	1,409 (3.58)^a^	181 (1.39)^a^	282 (2.24)^a^	268 (5.93)^a^	461 (18.03)^a^	< 0.001
4 (%)	1,548 (1.23)	65 (0.12)^a^	792 (2.01)^a^	141 (1.08)^a^	62 (0.49)^a^	179 (3.96)^a^	309 (12.08)^a^	< 0.001
5 (%)	673 (0.53)	46 (0.09)^b^	342 (0.87)^a^	59 (0.45)^b^	14 (0.11)^a^	84 (1.86)^a^	128 (5.01)^a^	< 0.001
**Healthcare utilisation 2019**								
GP visits (mean (SD))	4.21 (2.87)	2.27 (1.84)^a^	5.87 (2.43)^b^	4.56 (2.60)^a^	5.99 (2.77)^b^	5.82 (2.82)^a^	6.34 (3.06)^a^	< 0.001
General specialist visits (mean (SD))	2.70 (3.15)	1.42 (1.92)^a^	2.59 (2.42)^a^	3.00 (2.77)^a^	7.57 (4.31)^a^	4.18 (3.66)^b^	3.26 (3.45)^b^	< 0.001
Specialised specialist visits (mean (SD))	0.62 (1.32)	0.14 (0.44)^a^	0.42 (0.68)^a^	0.46 (0.83)^a^	3.17 (2.21)^b^	0.96 (1.52)^a^	1.48 (2.19)^b^	< 0.001
Emergency visits (mean (SD))	0.21 (0.57)	0.01 (0.08)^a^	0.04 (0.20)^a^	1.41 (0.74)^a^	0.18 (0.45)^a^	0.28 (0.62)^a^	1.01 (1.19)^a^	< 0.001
Non-elective inpatient admissions (mean (SD))	0.10 (0.41)	0.01 (0.11)^a^	0.07 (0.25)^a^	0.10 (0.30)^b^	0.09 (0.29)^a^	0.16 (0.42)^a^	2.22 (1.19)^b^	< 0.001
Elective inpatient admissions (mean (SD))	0.11 (0.41)	0.03 (0.18)^a^	0.05 (0.24)^a^	0.15 (0.43)^a^	0.32 (0.61)^a^	0.38 (0.72)^a^	0.98 (1.38)^a^	< 0.001
Ambulatory hospital visits (mean (SD))	0.19 (0.70)	0.03 (0.19)^a^	0.05 (0.22)^a^	0.13 (0.39)^a^	0.19 (0.47)^a^	3.19 (1.25)^a^	0.60 (1.12)^a^	< 0.001

^a^Significantly different from all five segments

^b^Significantly different from four other segments

All at 0.05/5 = 0.01 significance level (Bonferroni correction). All variables are significantly different across segments at a < 0.001 significance level using ANOVA or Kruskal–Wallis test

*Abbreviations*: *GP* general practitioner, *SD* standard deviation

Segment one “Low overall care use” included not only most of the study population (42.89%) but also the youngest patients (mean 37.76, SD 13.02), followed by segment three “High emergency care use” (mean 41.24, SD 17.02). The oldest patients were in segment six “High overall care use” (mean 69.01, SD 18.59), making up the smallest segment with only 2.03% of the population. In addition, the differences in the variables gender, residential care status and long-term care grade were also statistically significant with *p* < 0.001 (Table 2). While only 0.20% of the population in segment one lived in residential care homes, 14.94% of the population in segment six did so. Moreover, more than half of the population in segment six had a long-term care grade (54.40%).

The disease patterns were also found to differ between the segments (Table 3). The most frequent comorbidity listed in the Charlson index in the study population was chronic pulmonary disease (15.45%), followed by diabetes without chronic complications (11.44%) and congestive heart failure (8.34%). The prevalence of most of the comorbidities was lower than population average in segments one and three and higher than population average in the other segments. With 99.36%, most people living with a moderate or severe liver disease were in segment five. For this disease, segment five is the only segment with a prevalence higher than population average.

**Table 3:** Disease patterns

	**All**	**Segment 1: low overall care use**	**Segment 2: high primary care use**	**Segment 3: high emergency care use**	**Segment 4: high specialist care use**	**Segment 5: high hospital care use**	**Segment 6: high overall care use**	***p*-value**
**Prevalence of comorbidities**								
Myocardial infarction (%)	3,147 (2.50)	89 (0.16)^a^	1,605 (4.08)^a^	196 (1.50)^a^	725 (5.76)^a^	140 (3.10)^a^	392 (15.33)^a^	< 0.001
Congestive heart failure (%)	10,513 (8.34)	247 (0.46)^a^	5,764 (14.66)^a^	549 (4.21)^a^	2,346 (18.65)^a^	526 (11.64)^a^	1,081 (42.28)^a^	< 0.001
Peripheral vascular disease (%)	7,602 (6.03)	196 (0.36)^a^	3,881 (9.87)^b^	388 (2.98)^a^	1,888 (15.01)^b^	453 (10.03)^a^	796 (31.13)^a^	< 0.001
Cerebrovascular disease (%)	8,536 (6.77)	245 (0.45)^a^	4,660 (11.86)^b^	473 (3.63)^a^	1,890 (15.03)^b^	496 (10.98)^a^	772 (30.19)^a^	< 0.001
Dementia (%)	2,755 (2.19)	18 (0.03)^a^	1,619 (4.12)^a^	213 (1.63)^b^	225 (1.79)^a^	312 (6.91)^a^	368 (14.39)^b^	< 0.001
Chronic pulmonary disease (%)	19,478 (15.45)	4,600 (8.51)^a^	7,110 (18.09)^a^	2,246 (17.24)^b^	3,828 (30.43)^b^	914 (20.23)^b^	780 (30.50)^b^	< 0.001
Rheumatologic disease (%)	4,222 (3.35)	238 (0.44)^a^	1,806 (4.59)^b^	238 (1.83)^a^	1.473 (11.71)^b^	251 (5.56)^a^	216 (8.45)^a^	< 0.001
Peptic ulcer disease (%)	1,184 (0.94)	145 (0.27)^a^	456 (1.16)^c^	137 (1.05)^b^	203 (1.61)^c^	78 (1.73)^a^	165 (6.45)^b^	< 0.001
Mild liver disease (%)	7,948 (6.31)	993 (1.84)^a^	3,725 (9.48)^b^	602 (4.62)^a^	1,671 (13.29)^b^	487 (10.78)^a^	470 (18.38)^a^	< 0.001
Diabetes without chronic complication (%)	14,417 (11.44)	725 (1.34)^a^	8,343 (21.23)^a^	963 (7.39)^b^	2,699 (21.46)^b^	810 (17.93)^a^	877 (34.30)^a^	< 0.001
Diabetes with chronic complication (%)	7,490 (5.94)	144 (0.27)^a^	4,384 (11.15)^a^	390 (2.99)^a^	1,558 (12.39)^a^	406 (8.99)^a^	608 (23.78)^a^	< 0.001
Hemiplegia or paraplegia (%)	2,278 (1.81)	191 (0.35)^a^	1,034 (2.63)^a^	197 (1.51)^b^	321 (2.55)^b^	238 (5.27)^a^	297 (11.62)^a^	< 0.001
Renal disease (%)	10,009 (7.94)	385 (0.71)^a^	5,506 (14.01)^b^	514 (3.94)^a^	1,979 (15.73)^b^	571 (12.64)^a^	1,054 (41.22)^a^	< 0.001
Any malignancy, including leukaemia and lymphoma (%)	8,009 (6.35)	427 (0.79)^a^	3,288 (8.36)^a^	367 (2.82)^a^	2,235 (17.77)^a^	1,023 (22.64)^a^	669 (26.16)^a^	< 0.001
Moderate or severe liver disease (%)	4,692 (3.72)	7 (0.01)^a^	68 (0.17)^b^	24 (0.18)	37 (0.29)^c^	4,489 (99.36)^a^	67 (2.62)^c^	< 0.001
Metastatic solid tumour (%)	1,740 (1.38)	50 (0.09)^a^	494 (1.26)^a^	67 (0.51)^a^	505 (4.01)^a^	334 (7.39)^a^	290 (11.34)^a^	< 0.001
AIDS/HIV (%)	90 (0.07)	20 (0.04)	38 (0.10)^b^	9 (0.07)	5 (0.04)	16 (0.35)	2 (0.08)	< 0.001
**Charlson Comorbidity index**								
0 (%)	74,959 (59.47)	46,506 (86.03)^a^	14,740 (37.50)^a^	8,598 (65.99)^a^	3,264 (25.95)^a^	1,522 (33.69)^a^	329 (12.87)^a^	< 0.001
1–2 (%)	37,601 (29.83)	7,412 (13.71)^a^	17,727 (45.10)^c^	3,738 (28.69)^b^	5,914 (47.02)^b^	2,071 (45.84)^b^	739 (28.90)^b^	< 0.001
3–4 (%)	10,247 (8.13)	131 (0.24)^a^	5,539 (14.09)^a^	539 (4.14)^a^	2,487 (19.77)^a^	734 (16.25)^a^	817 (31.95)^a^	< 0.001
> = 5 (%)	3,239 (2.57)	7 (0.01)^a^	1,301 (3.31)^b^	155 (1.19)^a^	913 (7.26)^b^	191 (4.23)^a^	672 (26.28)^a^	< 0.001

^a^Significantly different from all five segments

^b^Significantly different from four other segments

^c^Significantly different from three other segments

All at 0.05/5 = 0.01 significance level (Bonferroni correction). All variables are significantly different across segments at a < 0.001 significance level using Chi-square test

More than half of the study population (59.47%) had a Charlson index of 0, mostly documented in segments one and three. Only 2.57% of the population had a Charlson index of 5 or higher which mostly appeared in segments two, four, five and six, showing the tendency toward multimorbidity in these segments.

### Healthcare utilisation and costs

**Fig. 1:**
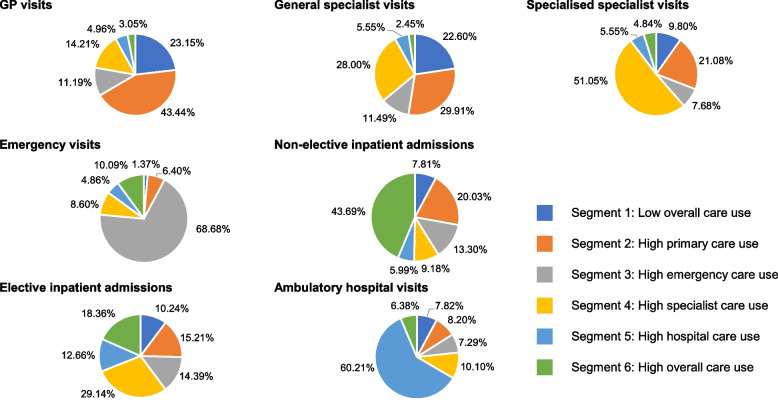
Utilisation for each type of health service by segment. Abbreviations: GP, general practitioner

**Fig. 2:**
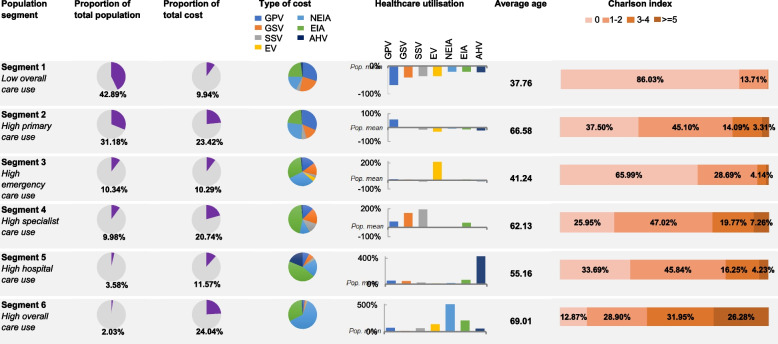
Overview over characteristics of segments adapted from [5, 6]. Abbreviations: GPV, GP visits; GSV, general specialist visits; SSV, specialised specialists visits; EV, emergency visits; NEIA, non-elective inpatient admissions; EIA, elective inpatient admissions; AHV, ambulatory hospital visits; Pop, population

The proportion of healthcare utilisation by each segment is presented in Fig. 1. All utilisation variables differed significantly across the segments with *p* < 0.001. Pairwise comparison between the segments demonstrated that for all the variables, this difference existed across all other segments or four other segments. Overall, the segments showed distinct characteristics (Fig. 2).

Segment one consisted of patients with a low overall care use. Not only were they younger than the population mean, but they also used less healthcare services in all types of healthcare settings assessed, whereas segments two to four all had an above-average use of certain healthcare settings. Similar to segment one, patients in segment two had a lower healthcare utilisation in almost every type of healthcare setting except for GP practice visits, which correlated with older age and higher prevalence of comorbidities in this segment (Tables 2 and 3). These were the two largest segments with about three quarters of the study population. Together, they accounted for 33.37% of total cost.

The patients in segment three were similar in age to those in segment one and had fewer healthcare provider visits than the average population when it came to specialised care practice visits and ambulatory hospital visits. However, their number of GP practice visits, general specialist care practice visits and elective inpatient admissions exceeded those of segment one. They were characterised by a high number of emergency care visits compared to the population mean (68.68% of all emergency visits) and accounted for 10.29% of total cost.

In segment four, patients were older, the number of GP practice visits, general specialist care practice visits, specialised care practice visits and elective inpatient admissions were higher than the population mean, but lower for emergency visits and non-elective inpatient admissions. This correlated with a higher prevalence of comorbidities. Overall costs of this segment comprised 20.74% of the population total cost.

For segments five and six, healthcare utilisation in all settings was higher than the population mean. In detail, segment five had the highest number of ambulatory hospital visits among all segments with 60.21% of all visits. Segment six is characterised by a very high overall healthcare utilisation, with GP practice visits, non-elective inpatient admissions and elective inpatient admissions being the highest among all segments. Covering only 2.03% of the study population, the segment accounted for 24.04% of the total cost. Both segments five and six showed a high rate of comorbidities.

## Discussion

This study applied a data-driven utilisation-based cluster analysis on a population in Germany. We were able to include all people insured with the AOK Baden-Wuerttemberg, which covered more than half of the population in the defined region, regardless of whether they had an encounter with the health system. Addressing the gap left by other studies that only included patients who utilised services in institutions belonging to a regional health system [8], or focused on patients registered with GPs [5, 6], this study considered healthcare utilisation of insureds outside the defined region across different healthcare settings. By doing so, patients with distinct healthcare needs and a use of health services outside the region who might have been previously excluded could be taken into account. The cluster analysis revealed six homogenous population segments. All segments were distinct in terms of demographics, morbidity and patterns of healthcare utilisation. This is in line with previous studies that applied the same segmentation method [5, 6, 8]. The number of segments derived in these studies ranged from five [8] to ten [6] cluster solutions, depending on the desired number of indistinguishable entities, and identified various types of low- and high-needs across the utilisation spectrum and across diseases.

The largest proportion of the population (42.89%) could be considered healthy with low overall care use. In previous studies this proportion varied between 38.0% [5] and 60.2% [6]. Despite the size of this group, patients’ needs are often overlooked in health care as this low-need population has little demand in services. Since remuneration of providers in the statutory health insurance in Germany is mainly based on a fee-for-service system combined with quarterly contact capitations [9], especially fee-for-service payments may lack incentives for prevention [26, 27]. In order to preserve patients’ health status, interventions should include disease prevention and health promotion as provided for in the German Act to Strengthen Health Promotion and Disease Prevention of 2015 [28]. Since people with no or only sporadic contact with the health system are also included, it is important to reach them additionally through employers (e.g., physical activation [29] or (diabetes) prevention programs [30]), communities (e.g., nutrition education [31]) or patient reminders and recall systems (e.g., immunisations [32] or cancer screening [33]).

The second-largest segment encompasses one third of the population and is similar to segment five by Vuik et al. [5] (“High primary care use”; 17%) and segment three by Low et al. [8] (“Stable, chronic disease”; 28.02%). It is characterised by patients with high primary care use and a higher prevalence of stable chronic conditions, i.e., without exacerbation (e.g., emergency department visit and hospitalisation). Healthcare strategies for segment two therefore should focus on promoting self-management, e.g., through telehealth technologies to obtain self-care skills and self-monitoring behaviours [34]. Additionally, case managers can be involved in healthcare coordination to educate and empower the patients according to their needs [35] to support physicians in providing patient care.

The high prevalence of emergency department visits combined with the second-lowest average age of 41.24 years and the second lowest Charlson comorbidity index in segment three could potentially be reduced by enhancing primary care management and extending practice hours [36]. Moreover, providing health education to individuals or informing them how to use the health system can reduce the use of emergency departments [37]. This study categorises 10.29% of the population in this segment. This is a higher rate compared to 9% (segment three “High emergency care use”) in Vuik et al. [5] and 5.4% (segment three “Low overall need, slightly higher use of emergency care”) in Nnoaham et al. [6], but in line with an overall increased number of especially ambulatory emergency department visits in Germany [38].

Segment four is characterised by high specialist care use and could benefit from better coordination of care within the fragmented German health system. Policymakers may need to strengthen frameworks that enable and support integrated care, while advanced practice nurses should take over tasks such as the management of chronic diseases or home visits to increase physician capacities [39]. The proportion of the population in this segment was 9.98%, a lower rate than the 14% (segment four “Specialist care use”) [5], 16.06% (segment four “Complicated chronic disease”) [8] and 16% (segments five, six, seven combined: “Low overall need, slightly higher use of primary and specialist care”, “High overall need, high use of primary and specialist care” and “High need due to a very high use of specialist care”) [6] reported by previous studies for a similar segment. Potential reasons include the German coding specifics and sectoral separation. Some of the visits classified as specialist visits internationally, may be counted as ambulatory hospital visits in the German health system and hence fall into segment five. Thus, health interventions and strategies must be developed in the light of the health system as they differ internationally.

Ambulatory hospital visits, which are unique to the German health system, are mostly documented in segment five. Patients in this segment (3.58%) have a higher number of elective inpatient admissions, like segment two (“High need due to very high elective admissions”; 5.9%) by Nnoaham et al. [6]. They should be targeted by utilisation management strategies and interventions such as pre-admission reviews [40].

The smallest segment six (2.03%) accounts with 24.04% for the highest proportion of total cost. It consists of the oldest patients with high needs and overall high healthcare use. As there is a high rate of comorbidities and malignancy in this segment, end-of-life care with its various patient-centred aspects of palliative or hospice care should be focused on [41]. In addition, it is crucial that patients are being supported in handling their conditions while preserving vitality [5]. Nevertheless, interventions should consider that about 15% in this segment live in residential care homes. Similar to this segment are the “Frequent admitters” (segment five; 1.79%) by Low et al. [8], although they are younger on average and have less visits in all healthcare settings but in specialist outpatient visits. Both Vuik et al. [5] and Nnoaham et al. [6] split the high-needs segments in several subgroups (segments six, seven and eight: “Very high needs and high emergency care use”, “High needs but low emergency care use” and “High needs, emergency and home care use” vs. segments four, six and nine: “Very high overall need, high use of emergency care”, High overall need, slightly higher use of specialist care” and “High overall need, high use of primary and emergency care”) with a prevalence of 22% vs. 5.1%, respectively.

Dividing a population into distinct and homogenous groups based on age and healthcare utilisation can support in identifying various low- and high-needs patients, even if age and utilisation are only proxies for the actual healthcare need of patients in the respective segments. Furthermore, the segments show the heterogeneity in healthcare requirements of different patient types. For example, while patients in segments two and five are similar in the count of comorbidities, patients in segment two are is older and have a lower than average utilisation of all healthcare settings except for primary care, whereas segment five uses all healthcare settings more often, especially hospital care. Overall, segmentation offers the opportunity to develop more effective healthcare concepts that target patient groups according to their healthcare needs.

### Limitations

There are several limitations when interpreting the results of this study. As health insurance claims data is collected routinely for billing purposes, only restricted information was available on the severity of the diseases and further determinants of health, such as the social-economic status. Future research should link data from different data sources to provide more detailed insights into the population needs. Also, this study is limited by using a proxy for the number of physician visits. In Germany, multiple doctor-patient contacts in one quarter are usually billed at the first visit in the quarter, therefore, the number of visits was assessed in a quarterly period underestimating the actual number of visits. Insureds have the opportunity to voluntarily enrol in GP-centred models of care (“Hausarztzentrierte Versorgung”, HZV), which requires them, for instance, to consult their GP first to get a referral for specialist care. The GP is the first point of contact for patients taking the role of a gatekeeper, which may lead to an increased number of GP visits but less uncoordinated encounters with specialists [42, 43]. The data used in this study was collected over a one-year period and might thereby be sensitive to random variation due to influenza waves, for example. Longitudinal data will be needed to give more robust information on the patient’s healthcare utilisation and disease patterns as well as to show patient movements between the respective segments. Another potential limitation is the limited generalisability of the results due to historically determined different structures of insureds in statutory health insurance funds in Germany, e.g., income and educational status may differ between health insurance funds [44]. Thus, including the whole population covered by various health insurance funds in further studies might lead to different segment sizes and segment types. Finally, the district presented in this study is an example of a rural region in Germany. Urban regions may show different healthcare utilisation patterns.

## Conclusion

Dividing a population into distinct and homogenous groups according to their healthcare needs is a powerful tool to understand the population’s demand for healthcare services in more depth. Using age and healthcare utilisation as segmentation variables supports the identification of low- and high-needs patients who differ in terms of their healthcare utilisation, morbidity, and demographic characteristics. Consequently, health policymakers and insurers can use population segmentation to align the planning of healthcare services with the healthcare needs of population groups to properly allocate healthcare resources, and finally to strengthen intersectoral health care and planning. Nevertheless, although data-driven population segmentation approaches can be adapted to different health systems, peculiarities of health systems such as coding practices must be considered to make valid use of segmentation approaches.

## Data Availability

The datasets used and analysed in the study are not publicly available but may be available from the AOK Baden-Wuerttemberg on reasonable request. Access to the data was obtained from the AOK Baden-Wuerttemberg under Data Disclosure Agreements.

## Abbreviations

AHV: Ambulatory hospital visits
EIA: Elective inpatient admissions
EV: Emergency visits
GP: General practitioner
GPV: General practitioner visits
GSV: General specialist visits
HZV: GP-centred model (“Hausarztzentrierte Versorgung “)
NEIA: Non-elective inpatient admissions
Pop: Population
SD: Standard deviation
SGB: Social Code Book (“Sozialgesetzbuch “)
SSV: Specialised specialists visits
